# Chemokine Analysis in Patients with Metastatic Uveal Melanoma Suggests a Role for CCL21 Signaling in Combined Epigenetic Therapy and Checkpoint Immunotherapy

**DOI:** 10.1158/2767-9764.CRC-22-0490

**Published:** 2023-05-18

**Authors:** Vasu R. Sah, Henrik Jespersen, Joakim Karlsson, Lisa M. Nilsson, Mattias Bergqvist, Iva Johansson, Ana Carneiro, Hildur Helgadottir, Max Levin, Gustav Ullenhag, Anders Ståhlberg, Roger Olofsson Bagge, Jonas A. Nilsson, Lars Ny

**Affiliations:** 1 Sahlgrenska Center for Cancer Research, Department of Surgery, Institute of Clinical Sciences, Sahlgrenska Academy, University of Gothenburg, Gothenburg, Sweden.; 2 Department of Oncology, Institute of Clinical Sciences, Sahlgrenska Academy at University of Gothenburg, Sahlgrenska University Hospital, Gothenburg, Sweden.; 3 Department of Oncology, Oslo University Hospital, Oslo, Norway.; 4 Harry Perkins Institute of Medical Research, University of Western Australia, Perth, Western Australia, Australia.; 5 Biovica International AB, Uppsala Science Park, Uppsala, Sweden.; 6 Department of Oncology, Sahlgrenska University Hospital, Gothenburg, Sweden.; 7 Department of Oncology, Skåne University Hospital, Lund, Sweden.; 8 Department of Oncology, Karolinska University Hospital, Stockholm, Sweden.; 8 Department of Oncology, Sahlgrenska Hospital, Uppsala, Sweden.; 9 Department of Radiology, Oncology and Radiation Science, Section of Oncology, Uppsala University, Uppsala, Sweden.; 10 Department of Laboratory Medicine, Wallenberg Centre for Molecular and Translational Medicine, University of Gothenburg, Gothenburg, Sweden.; 11 Department of Clinical Genetics and Genomics, Sahlgrenska Center for Cancer Research, Institute of Biomedicine, University of Gothenburg and Sahlgrenska University Hospital, Gothenburg, Sweden.; 12 Department of Surgery, Sahlgrenska University Hospital, Gothenburg, Sweden.; 13 Wallenberg Centre for Molecular and Translational Medicine, University of Gothenburg, Gothenburg, Sweden.

## Abstract

**Purpose:**

Patients with metastatic uveal melanoma have limited therapeutic options and high mortality rate so new treatment options are needed.

**Patients and Methods:**

We previously reported that patients treated with the PD-1 inhibitor pembrolizumab and the histone deacetylase inhibitor entinostat in the PEMDAC trial, experienced clinical benefits if their tumor originated from iris or was wildtype for *BAP1* tumor suppressor gene. Here we present the 2-year follow-up of the patients in the PEMDAC trial and identify additional factors that correlate with response or survival.

**Results:**

Durable responses were observed in 4 patients, with additional 8 patients exhibiting a stable disease. The median overall survival was 13.7 months. Grade 3 adverse events were reported in 62% of the patients, but they were all manageable. No fatal toxicity was observed. Activity of thymidine kinase 1 in plasma was higher in patients with stable disease or who progressed on treatment, compared with those with partial response. Chemokines and cytokines were analyzed in plasma. Three chemokines were significantly different when comparing patients with and without response. One of the factors, CCL21, was higher in the plasma of responding patients before treatment initiation but decreased in the same patients upon treatment. In tumors, CCL21 was expressed in areas resembling tertiary lymphoid structures (TLS). High plasma levels of CCL21 and presence of TLS-like regions in the tumor correlated with longer survival.

**Conclusions:**

This study provides insight into durable responses in the PEMDAC trial, and describes dynamic changes of chemokines and cytokines in the blood of these patients.

**Significance:**

The most significant finding from the 2-year follow-up study of the PEMDAC trial was that high CCL21 levels in blood was associated with response and survival. CCL21 was also expressed in TLS-like regions and presence of these regions was associated with longer survival. These analyses of soluble and tumor markers can inform on predictive biomarkers needing validation and become hypothesis generating for experimental research.

## Introduction

Immunotherapy with immune checkpoint inhibitors (ICI) blocking CTLA4 and PD-1 has revolutionized the treatment of metastatic cancers such as melanoma (1), kidney cancer (2), and lung cancer (3). In some of these patients, durable responses can last for years, with acceptable levels of toxicities compared with standard chemotherapy (4–6). However, in some patients, disease progression continues, despite the start of treatment, or it resumes progression after an initial response. In addition, some of these patients experience severe toxicities (7, 8). To address these issues, intense work is ongoing to understand treatment resistance and how to manage and reduce adverse effects (9). Today, a multitude of trials are combining ICIs with chemotherapy (10), radiotherapy (11), vaccines (12), targeted therapies (13), cellular therapies (14), and more (15). Thus far, in melanoma, combination therapies have been shown to be more effective than monotherapy with anti-PD-1 inhibitors, but only additively (16).

Uveal melanoma (UM) is a rare form of melanoma that arises in the uvea (choroid, ciliary body, and the iris) of the eye (17). The local disease can be managed by brachytherapy or enucleation, which can be curative. Unfortunately, half of all patients with uveal melanoma develop metastases, most often to the liver (18). Metastatic uveal melanoma has a very poor prognosis. In a nonselected population, median overall survival (OS) is around 1 year and progression-free survival (PFS) is just 3 months (19). Recently, three phase II studies and one phase III trial have demonstrated longer survival than historical controls. The T-cell engager tebentafusp (20) demonstrated a doubling of OS in a phase III trial, without improving PFS. However, this treatment only works for patients with HLA-A2 genotype. A recently reported phase III trial randomized patients to either isolated hepatic perfusion (IHP) or best alternative care (BAC). IHP showed an overall response rate of 40% compared with 4.5% in the BAC group and a PFS of 4.1 months compared with 2.1 months for the BAC group (21). Two phase II trials combining the CTLA4 inhibitor ipilimumab and the PD-1 inhibitor nivolumab (22, 23) and one phase II trial combining the PD-1 inhibitor pembrolizumab and the histone deacetylase (HDAC) inhibitor entinostat (PEMDAC; ref. 24) demonstrated around 2 months extended OS.

The rationale behind the PEMDAC trial was the finding that HDAC inhibition can enhance immunogenicity of therapy-resistant melanoma, including uveal melanoma (25). The trial met its primary endpoint of objective response rate (ORR) by a small margin. Four partial responses (PR) were observed (24). Sequencing suggested that if the tumor was wildtype for *BAP1* or a UV-damaged iris melanoma, then the patient was more likely to respond and have a more favorable survival. Low base line circulating tumor DNA (ctDNA) or lactate dehydrogenase (LDH) also predicted longer survival, suggesting that the level of tumor burden was associated with the ability of the immune system to tackle the disease. Immune profiling also suggested that elevated levels of activated T cells resulted in longer survival (24).

The outcomes of current combination therapy trials provide clinical rationale for continuing pursuing immunotherapy for metastatic uveal melanoma, with the aim to improve immunity against uveal melanoma and responses in patients. A key aspect that needs further study is the long-term follow-up of patients receiving these therapies and identifying novel biomarkers that can help researchers predict patient responses to treatments. On the long term, identifying these biomarkers may help with the design of more efficient therapies for uveal melanoma with fewer side effects.

The aim of this study is to follow-up patients in the PEMDAC trial and identify additional potential blood and tissue correlates of response or survival. Here we present 2-year survival data and present novel data on the utility of circulating thymidine kinase 1 (TK1) and chemokines as potential biomarkers to understand responses in the PEMDAC trial.

## Materials and Methods

### Clinical Trial and Design

The clinical study protocol and all amendments were approved by the Swedish Medical Product Agency (EudraCT registration number: 2016-002114-50) as well as the Regional Ethical Review Board at the University of Gothenburg (Gothenburg, Sweden; dnr 692-16). The study was conducted in accordance with the International Conference on Harmonization - Good Clinical Practice guidelines, and the Declaration of Helsinki. All patients provided oral and written informed consent before inclusion. The study is registered March 3, 2016, at ClinicalTrials.gov. number: NCT02697630.

The trial was designed as a phase II, single-arm, multicenter study and is an investigator-initiated trial within the Merck Investigator Study Program. The study was carried out at the four major Swedish university hospitals with support of the Swedish Melanoma Study Group. Patients received pembrolizumab 200 mg intravenously every third week in combination with entinostat at a starting dose of 5 mg orally once weekly. Dose reduction of entinostat was allowed according to prespecific criteria for hematologic toxicity. Treatment continued until disease progression, intolerable adverse reactions, patient's withdrawal of consent, or decision of the investigating physician to end treatment, or to a maximum period of 2 years of treatment. Efficacy and safety were assessed in all allocated patients who received ≥1 dose of study treatment. Clinical efficacy was assessed according to RECIST, version 1.1. Adverse events (AE) and laboratory abnormalities were collected during study treatment and graded according to the NCI Common Terminology Criteria for Adverse Events, version 4.0 and further classified using the MEDRA system.

The primary endpoint was ORR (proportion of patients with complete response or PR). Secondary endpoints included clinical benefit rate at 18 weeks after start of treatment, PFS, OS, and safety.

### Patients

Eligibility criteria are available in the clinical study protocol which has been published (26). Key criteria for inclusion were age ≥18 years, histologically or cytologically confirmed diagnosis of metastatic uveal melanoma, measurable disease according to RECIST 1.1, and Eastern Cooperative Oncology Group performance status 0–1. Both treatment-naïve patients and previously treated patients were allowed to participate. Exclusion criteria included patients with active brain metastases, autoimmune disease, ongoing treatment with systemic corticosteroids (above 10 mg prednisolone), or previous treatment with anticancer immunotherapy. The first patient included in the study was enrolled in February 2018 and the last patient was enrolled in December 2018. A sample size of 29 patients was planned, allocated using Simon's optimal two-stage design. At least one confirmed response in the first 10 patients was required to continue enrollment for an additional 19 patients. Patient representativeness is available in Supplementary Table S1.

### Plasma Samples and Biobanking

Whole blood was collected in EDTA tubes and centrifuged at room temperature within 2 hours at 2000 × *g* for 10 minutes. Plasma was collected for upto a period of 2 years and stored at −80°C. All samples from four clinical trial units were collected together and biobanked at Sahlgrenska Center for Cancer Research for further exploratory analysis.

### TK Activity Level Analysis

Plasma TKa levels were determined using the DiviTum TKa assay (Biovica) in accordance with the manufacturer's instructions. DiviTum TKa is a refined ELISA-based test reflecting cell proliferation rate by measuring TKa in serum, plasma, or cells (27). In summary, plasma was mixed with the reaction mixture in a 96-well ELISA plate, and bromodeoxyuridine (BrdU) monophosphate was generated by TK reaction, phosphorylated to BrdU triphosphate, and incorporated into a synthetic DNA strand. An anti-BrdU mAb conjugated to the enzyme alkaline phosphatase and a chromogenic substrate were used to detect BrdU incorporation. The absorbance readings were converted using standards with known TKa values (working range from 100 to 2,000 DuA). The lower limit of detection of the assay is set at 100 DuA, and all values below the threshold were reported as <100 DuA. The DuA value is a combination of TK specific activity multiplied by the amount of TK protein concentration (pg/mL).

### Cytokine and Chemokine Measurements

In this study, we used Luminex xMAP technology for multiplexed quantification of 71 Human cytokines, chemokines, and growth factors. The multiplexing analysis was performed using the Luminex 200 system (Luminex) by Eve Technologies Corp. Seventy-one markers were simultaneously measured in the samples using Eve Technologies’ Human Cytokine 71-Plex Discovery Assay which consists of two separate kits; one 48-plex and one 23-plex (MilliporeSigma). The assay was run according to the manufacturer's protocol. The 48-plex consisted of sCD40L, EGF, Eotaxin, FGF-2, FLT-3 Ligand, Fractalkine, GCSF, GMCSF, GROα, IFNα2, IFNγ, IL1α, IL1β, IL1RA, IL2, IL3, IL4, IL5, IL6, IL7, IL8, IL9, IL10, IL12 (p40), IL12(p70), IL13, IL15, IL17A, IL17E/IL25, IL17F, IL18, IL22, IL27, IP-10, MCP-1, MCP-3, MCSF, MDC, MIG/CXCL9, MIP-1α, MIP-1β, PDGF-AA, PDGF-AB/BB, RANTES, TGFα, TNFα, TNFβ, and VEGF-A. The 23-plex consisted of 6CKine, BCA-1, CTACK, ENA-78, Eotaxin-2, Eotaxin-3, I-309, IL16, IL20, IL21, IL23, IL28A, IL33, LIF, MCP-2, MCP-4, MIP-1δ, SCF, SDF-1α+β, TARC, TPO, TRAIL, and TSLP. Assay sensitivities of these markers range from 0.14 to 55.8 pg/mL for the 71-plex. Individual analyte sensitivity values are available in the MILLIPLEX protocol.

### Follow-up

The patients had a minimum follow-up of 24 months with a last patient last visit in December 2020 for the active treatment phase. This analysis refer to a database lock December 20, 2020 to which all clinical efficacy and safety data refer to if not otherwise stated.

The patients were grouped on the basis of TK1 and cytokine/chemokine levels in plasma before start of the treatment, and observed for PFS, and OS. PFS was defined as the time from start of treatment until the date of confirmed progression or the date of death or of the last follow-up. OS was defined from the start of treatment until the date of death or last follow-up. All patients were analyzed for TKa and cytokine/chemokine levels from start of treatment followed by every 3-week timepoints, until the date of death or last follow-up.

### RNA sequencing

RNA were prepared from formalin-fixed, paraffin-embedded (FFPE) sections from patients in the PEMDAC trial using the Tissue FFPE DNA/RNA kit (Qiagen), with exome and RNA sequencing (RNA-seq) performed at the Genome Medicine Center at Sahlgrenska University Hospital, Gothenburg, Sweden.

### Differential Gene Expression Analysis

Preprocessed RNA-seq data, aligned to the 1000 Genomes (28) version of the hg19 human reference genome (v.37) with STAR (29) and quantified at gene level with htseq-count (HTSeq v. 0.11.2; ref. 30), from a previous study on the same subjects (24) were used to test for differential expression between long-term and short-term surviving patients with DESeq2 (v. 1.34.0) in R (v. 4.1.0). For this, a design taking into account batch, sex, anatomic tumor biopsy site and survival category was used, to account for confounding factors. A value of alpha = 0.05 was used with the DESeq “results” function and *P* values were adjusted were adjusted with the default Benjamini–Hochberg correction.

### Flow Cytometry

Flow cytometry from peripheral blood mononuclear cells were analyzed by Clinical Immunology Center, Sahlgrenska University Hospital Gothenburg, Sweden. Phenotyping was performed for CD3 (Pacific Blue), CD4 (PerCP-Cy5.5), CD8 (APC-Cy7), CD38 (APC), CD45RA (PE-Cy7), CCR7 (PE), CD31 (FITC), and HLA-DR (Am-Cyan) between patient response groups.

### IHC Analyses

FFPE baseline tumor samples were evaluated using hematoxylin and eosin stain and IHC. IHC was performed with an autostainer (Autostainer Link 48, Dako) using primary antibodies SOX10 (E6B6I, Cell Signaling Technology), TK1 (PA5-29686, Thermo Fisher Scientific), CCL21 (NBP2-37928, Novus), CCR7 (EPR23192-57, Abcam), and CD20 (Dako Clinical grade) antibodies. HRP Magenta (DAKO) was used to stain the protein of interest and counterstaining was done using hematoxylin.

### Statistical Analysis

Analyses of efficacy and safety in patients were carried out in all patients who received one dose of study treatment. The sample size and power estimation is based on the primary endpoint ORR, only. Power is required to be 80%. Significance is generally set to 5%. We assume that an ORR of 5% is not a clinically relevant treatment effect, whereas 20% is sufficient to consider the treatment useful. Enrollment was done according to Simon's optimal two-stage design [significance level = 5% (one-sided)] (Simon, 1989). The study was considered positive if at least 4 patients of the total of 29 have a confirmed objective response. Outcome measures that are proportions are reported using a 95% confidence interval (CI). Outcome measures are analyzed using nonparametric methods. Time is summarized using medians through the Kaplan–Meier method, together with 95% CIs. Details of the statistical analysis are described elsewhere (24, 26).

Chemokines were analyzed using two-tailed unpaired *t* tests between different response groups [progressive disease (PD), PR, and stable disease (SD)] of patients. In another analysis, chemokine levels in blood were compared between baseline, 9 weeks and end-of-study samples from each patient using two-tailed paired *t* tests. In these tests, *P* values were adjusted with FDR correction. Survival plots for PFS and OS were analyzed using log-rank (Mantel–Cox) test with GraphPad prism. All *P* values are represented as *, *P* < 0.05; **, *P* < 0.01; and ***, *P* < 0.001. All error bars represent SEs (SEM), unless otherwise stated.

### Data Availability

Sequencing data of this study have been deposited in European Genome-phenome Archive (EGA) with the accession code EGAS00001005478, under restrictions of controlled access. Chemokine data and flow cytometry data were generated by Eve Technologies Corp and the Clinical Immunology Center, Sahlgrenska University Hospital Gothenburg, Sweden, respectively. Derived data supporting the findings of this study are available from the corresponding author upon request.

## Results

### Two-year Follow-up of the PEMDAC Trial

**FIGURE 1:**
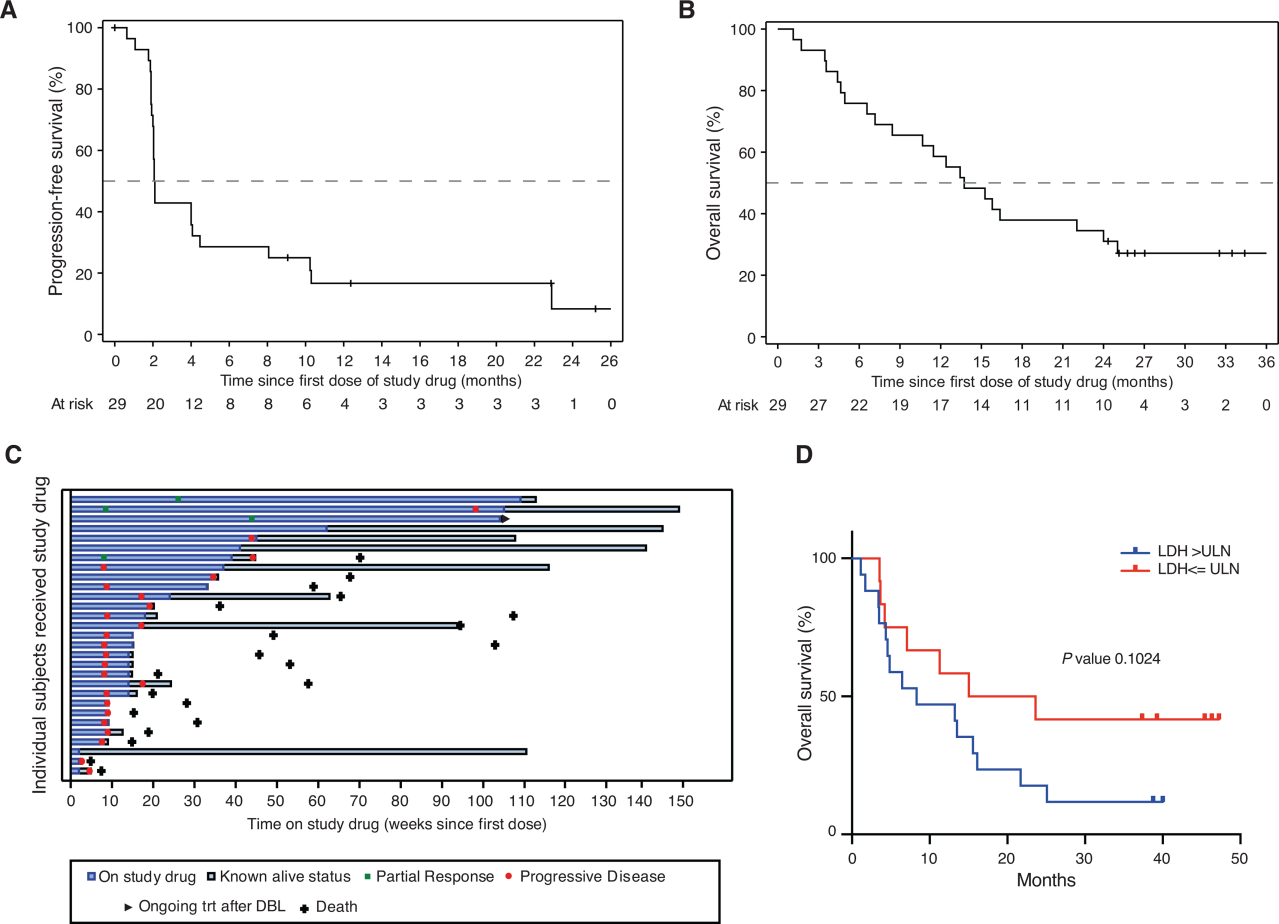
**A,** Kaplan–Meier analysis showing PFS of all patients. **B,** Kaplan–Meier analysis showing OS of all patients except one. **C,** Swimmer plot showing time on treatment, time to best response, and duration of response in all patients who received at least one dose of study drug (*n*  =  29) are shown. **D,** Kaplan–Meier analysis comparing OS between patients with LDH baseline greater or lower than the upper limit of normal (ULN).

Patient characteristics have been described elsewhere (24) and updates are provided as Supplementary Table S2. Median PFS and median OS remained similar at 2.1 months (95% CI, 2–4.1) and 13.7 months (95% CI, 7.2–22), respectively (Fig. 1A and B; Supplementary Fig. S1A and S1B). Twenty-eight percent (8/29) of the patients were still alive at database lock (DBL), that is, at least 2 years after start of treatment. In a *post hoc* analysis carried out for survival 6 months after DBL, 7 patients were still alive (Supplementary Table S3). Two patients had not received any subsequent therapy, whereas 5 had ongoing therapy. Of those 5, 3 were receiving chemotherapy and 2 immune checkpoint blockade (Fig. 1C).

Responses were observed in 4 patients in the PEMDAC trial, all of which lasted longer than 8 months (Fig. 1C; Supplementary Fig. S1B). However, levels of LDH, which could predict survival at the 1-year DBL, no longer significantly predicted survival (Fig. 1D). Safety remained similar to the 1-year analysis. AEs were reported in all 29 patients, and grade 3 toxicity was observed in 62% (18/29) patients (Supplementary Table S4). Immune-related grade 3 toxicity was reported in 34.5% (10/29) of patients. No quality-of-life measurements differed between before treatment and at last assessment (Supplementary Fig. S1C–S1E).

**FIGURE 2:**
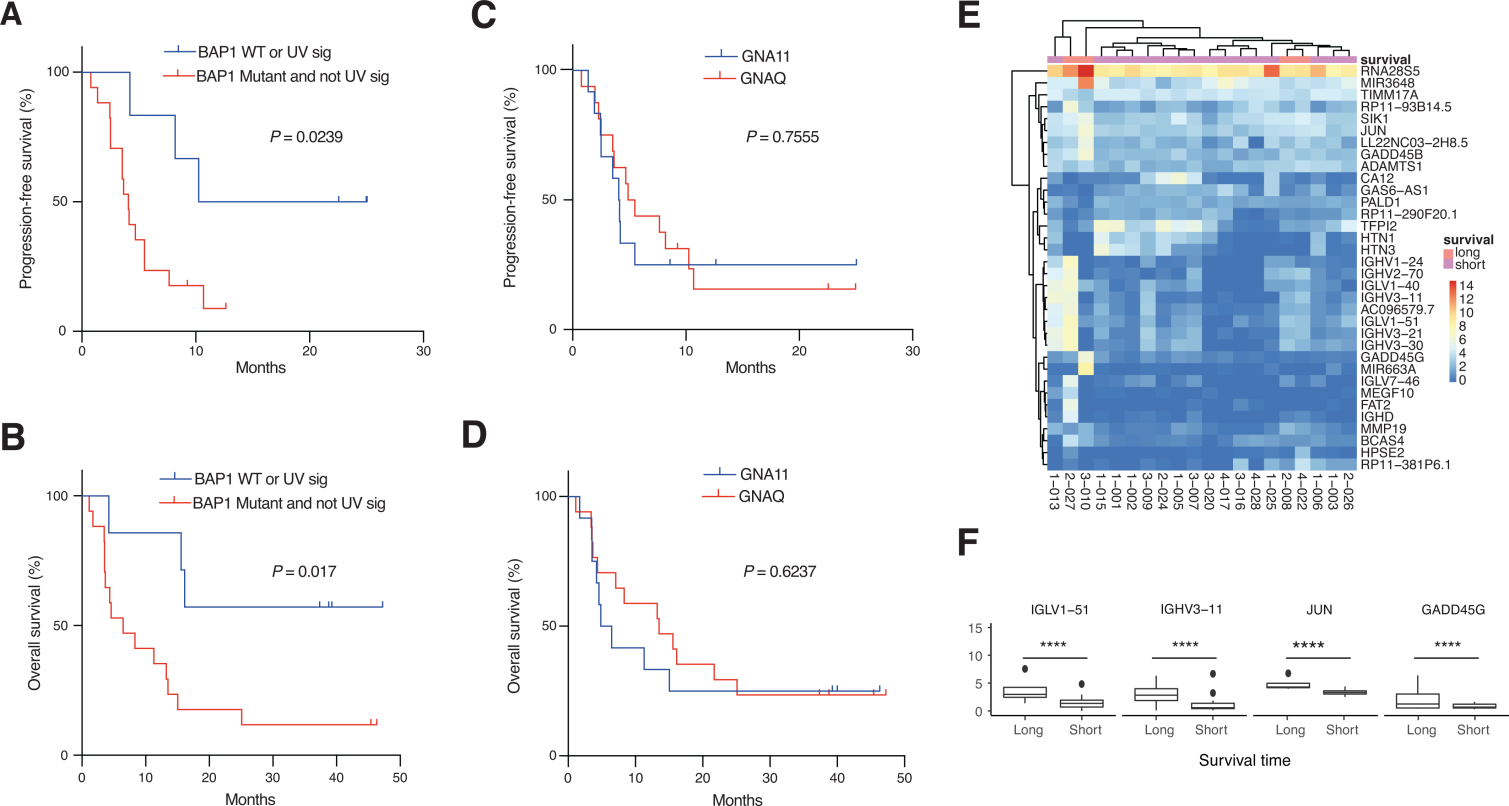
**A** and **B,** Kaplan–Meier analysis showing PFS and OS of patients with a wildtype *BAP1* status and UV-damaged uveal melanoma genome. PFS (**C**) and OS (**D**) analyses comparing patients with GNAQ- or GNA11-mutated uveal melanoma. **E,** Volcano plot showing differentially expressed genes between short term (*n* = 16) and long term (alive patients, *n* = 4) from bulk RNA-seq. Genes with FDR-adjusted *P* values <0.05 were considered statistically significant. **F,** Individual box plots showing relevant gene signatures implicating long-term (alive patients) survival. Statistical tests were carried out using DESeq2 and FDR-adjusted *P* values were denoted with *, *P* < 0.05; **, *P* < 0.01; ***, *P* < 0.001.

Genetic analysis confirmed our previous observation (24) that patients harboring a wildtype *BAP1* gene or who had a UV-damaged iris melanoma lived longer (Fig. 2A and B; Supplementary Table S3), regardless of their *GNAQ/GNA11* mutational status (Fig. 2C and D). We performed RNA-seq of pretreatment biopsies and investigated whether dichotomization of patients based on OS would identify any gene signature associated with survival. Unsupervised clustering did not cluster samples of long or short survival together, indicating there is no clear signature driving a difference in survival. However, some genes encoding either the light (e.g., *IGLV1-51*) or the heavy chain (*IGHV3-11*) of the B-cell receptor correlated with survival (Fig. 2E and F). Moreover, expression of genes known to be regulated by HDAC inhibitors such as *JUN* and *GADD45B*, *GADD45G* (31) were higher in patients with longer survival (Fig. 2E and F). In total, 34 genes were more highly expressed in patients with shorter survival after FDR correction (see Fig. 2E and F; Supplementary Table S5; Supplementary Fig. S2A). Gene set enrichment analysis revealed almost exclusively immune-related pathway gene sets including different Reactome pathways of B-cell receptor signaling (Supplementary Table S6).

### TK as a Biomarker of Response and Survival

TK1 is an enzyme involved in nucleotide biosynthesis and is elevated in many cancers, including melanoma (32). TK1 is a nuclear/cytoplasmic protein so just like LDH, it can leak out of cancer cells when they die (33). Thus, levels of TK1 in blood of patients are a correlate of tumor burden (34–36). This biomarker has previously been used in studies of metastatic cutaneous melanoma (37), but not uveal melanoma.

**FIGURE 3:**
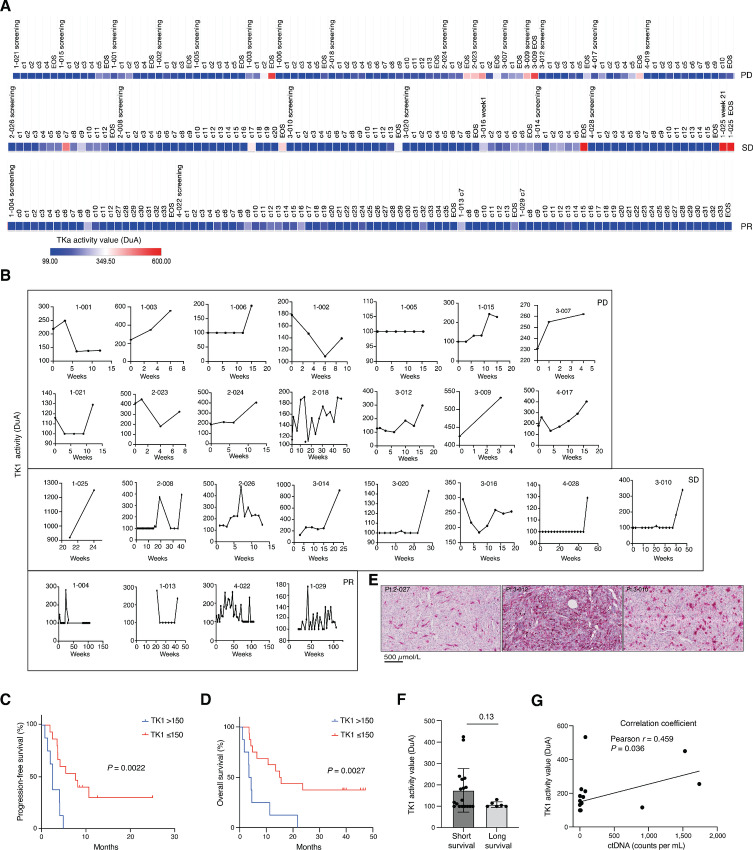
**A,** Heatmap showing TK1 activity value (DuA) from PEMDAC patient plasma samples, grouped within response groups. Each square is a timepoint for each patient and shows TK1 levels from pretreatment to end of study, until otherwise stated. Total plasma samples analyzed for TK1 = 287. **B,** Longitudinal TK1 activity for individual patients, as shown in A. **C** and **D,** Kaplan–Meier analysis showing PFS and OS, respectively for pretreatment TK1 values using a threshold of 150 DuA (median TK1 for all samples = 113). Patients with nonavailability of pretreatment samples were excluded from the analysis. **E,** IHC of TK1 showing nuclear/cytoplasmic magenta staining in patient biopsies 2-027, 3-012, and 3-010. **F,** Comparison between pretreatment TK1 values for short- and long-term survivors. **G,** Correlation between pretreatment TK1 (DuA) and circulating tumor DNA (counts/mL) matched patient samples (*n* = 21). All statistical tests were unpaired two-tailed *t* tests, assuming equal variance, with *, *P* < 0.05; **, *P* < 0.01; ***, *P* < 0.001.

We measured TK1 by its enzymatic activity in plasma from patients before or during treatment (Fig. 3A). We found that TK1 activity fluctuated significantly throughout this timeframe, and was detectable in all patients except one (Fig. 3B). Dichotomizing patients based on mean study TK1 activity and assessing survival, demonstrated that lower levels were associated with longer PFS and OS (Fig. 3C and D). IHC confirmed that TK1 was expressed in tumors (Fig. 3E). Dichotomizing patients on OS revealed that patients with longer survival had lower average levels of TK1 activity in blood than those with shorter survival (Fig. 3F). Numerically, PR patients exhibited lower levels of TK1 activity than patients with SD or PD (Supplementary Fig. S2B and S2C). TK1 levels weakly correlated with that of pretreatment ctDNA, which previously was shown to predict longer survival in the PEMDAC trial (Fig. 3G; ref. 24).

### Cytokines and Chemokines as Correlates of Response and Survival

**FIGURE 4:**
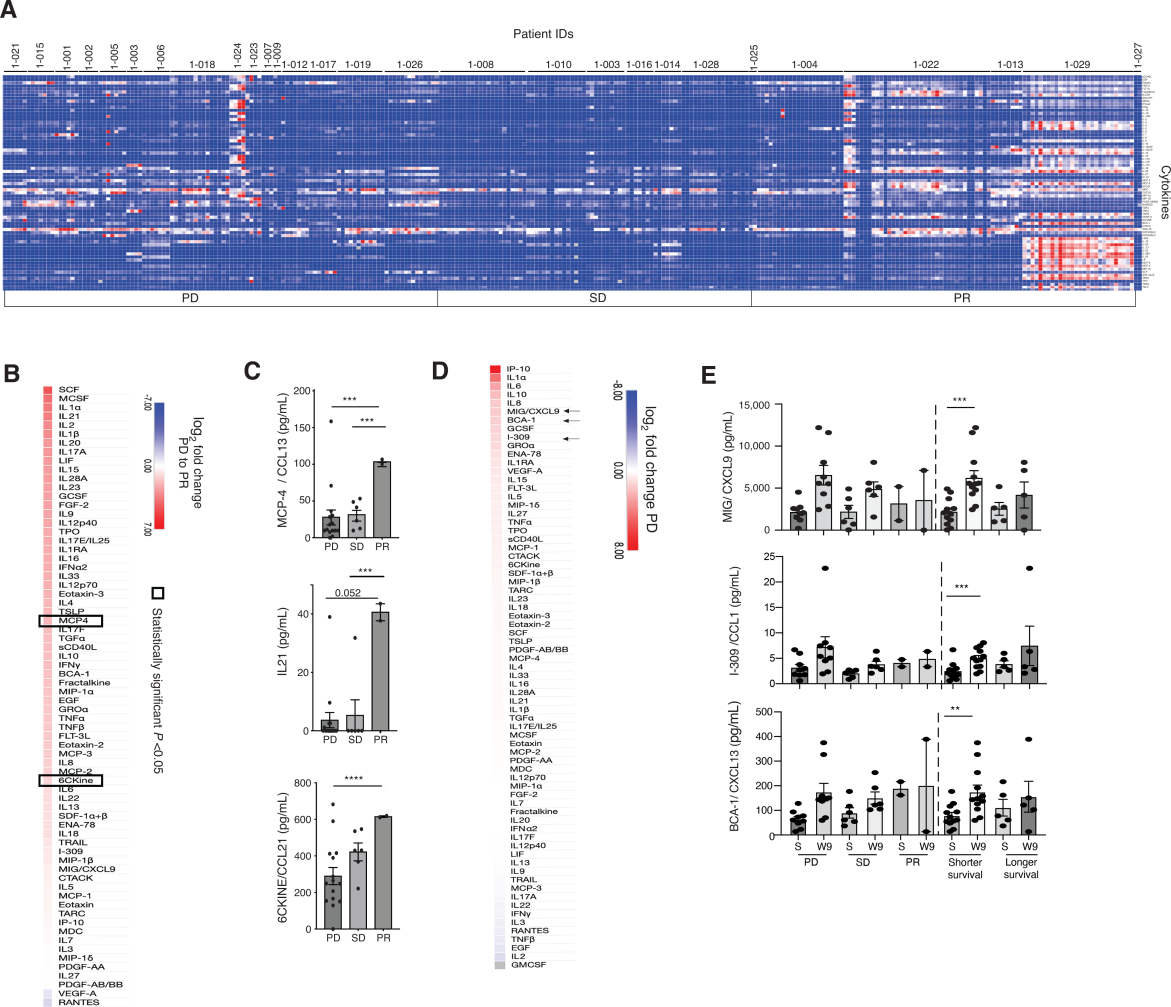
**A,** Heatmap of 71 chemokines and cytokines analyzed among all patients and their respective timepoints. Total plasma samples analyzed = 287. Each square represents a timepoint for each patient and shows response group-based levels from pretreatment until end of study, unless otherwise stated. **B,** Heatmap showing differential pretreatment values between PD and partial responders. Boxed chemokines are significant (*P*_adjusted_ < 0.05) after FDR correction. **C,** Individual chemokine or cytokine values (pg/mL) compared among different response groups. Only significantly different chemokines from B are included. **D,** Fold change difference between pretreatment values and week 9 after start of treatment are shown for PD patients. Arrows indicate chemokines that are significant (*P*_adjusted_ < 0.05) after FDR correction between patients that survived longer and those that survived shorter. **E,** Individual chemokine or cytokine values (pg/mL) compared among different response groups, only significant differences from D are included. Statistical tests in bar charts were unpaired two-tailed *t* tests (C), assuming unequal variance, or paired *t* tests (D) with *, *P* < 0.05; **, *P* < 0.01; ***, *P* < 0.001.

Next, we profiled 71 chemokine and cytokines using Luminex multiplex analysis. Dynamic changes where observed during treatment of chemokines and cytokines (Fig. 4A). Comparing pretreatment values between patients experiencing PD or a PR demonstrated three factors that had statistically different levels after FDR correction. These were higher in blood from patients with PR and included monocyte attractant CCL13 (38), inflammatory migration factors CCL21 (39), and inflammatory cytokine IL21 (ref. 40; Fig. 4B and C). Comparing pretreatment values and week 9 after start of treatment showed that most factors were reduced in the patients with. Three factors were elevated by treatment in patients with SD and/or PD after FDR correction (Fig. 4D and E). These included the T-cell exhaustion chemokine CXCL13 (41). The chemotactic chemokine CXCL9, was elevated in patients with SD and/or PD also when comparing with the end-of-study sample (Supplementary Fig. S3A and S3D).

**FIGURE 5:**
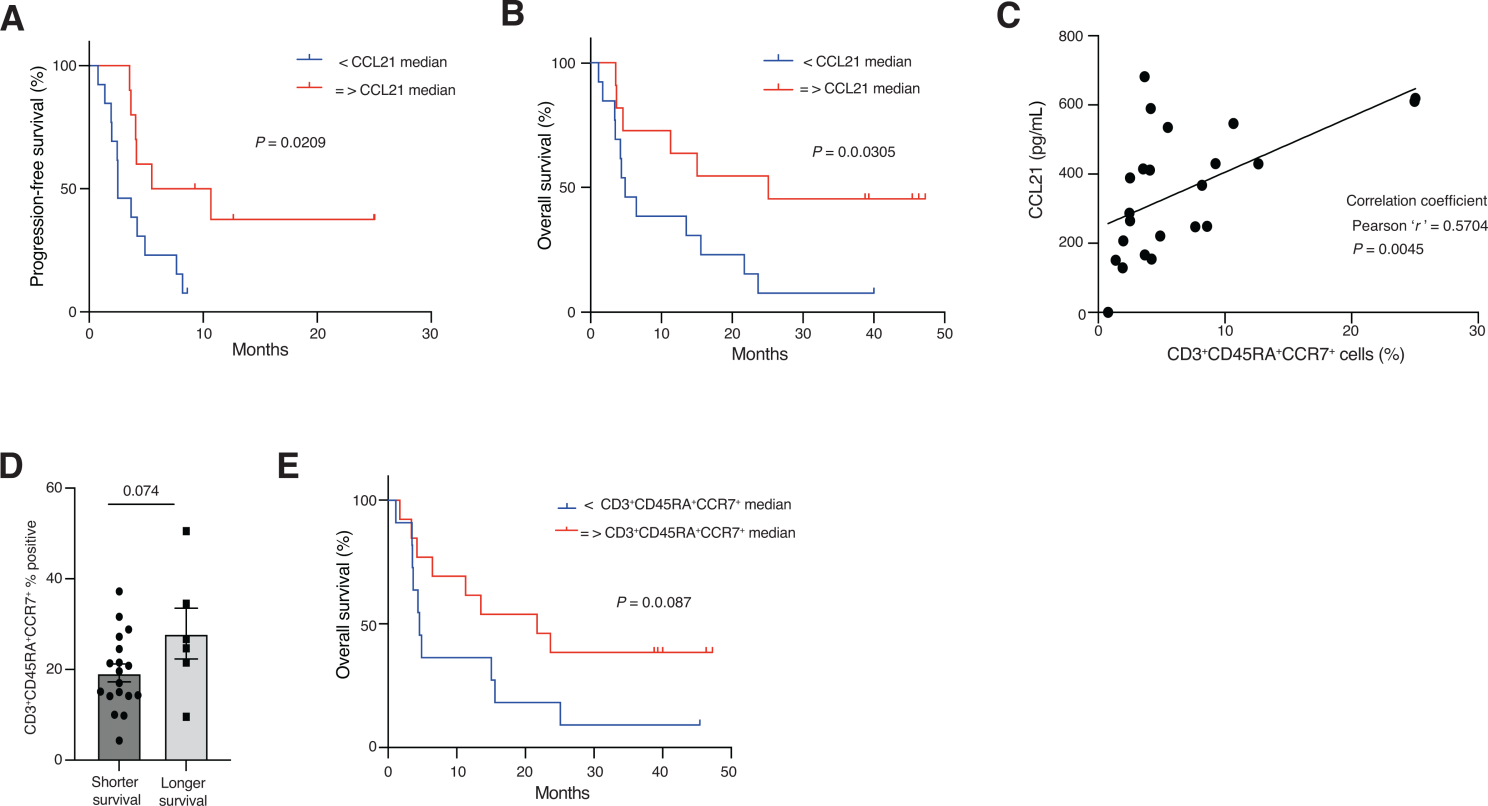
Kaplan–Meier analysis showing. PFS (**A**) and OS (**B**), respectively using pretreatment plasma CCL21 (pg/mL) measurements based on their median values. **C,** Correlation between CCL21 (pg/mL) and CD3^+^CCR7^+^CD45RA^+^ (% counts) matched patient samples (*n* = 23). **D,** Flow cytometry–based comparison between short- and long-term survivors for CD3^+^CCR7^+^CD45RA^+^ % (T naïve and T stem cell memory) analysis using pretreatment blood samples (*n* = 24). Statistical test was unpaired two-tailed *t* tests, assuming equal variance. **E,** Kaplan–Meier analysis showing OS using median CD3^+^CCR7^+^CD45RA^+^ % values.

CCL21 is a factor released by venule endothelial cells (42, 43) of inflammatory sites including tumors (39, 44) to allow T-cell migration and homing (45–48). To investigate whether CCL21 levels correlated with survival, we dichotomized survival data based on CCL21 levels. Both PFS and OS were longer in patients with higher plasma levels of CCL21 (Fig. 5A and B), but not levels of CCL13 or IL21 (Supplementary Fig. S4A–S4C). Plasma levels of CCL21 also correlated with number of CCR7^+^CD45RA^+^ T cells (Fig. 5C). We also interrogated whether expression on T cells of CCR7, the receptor for CCL21 (42, 49), would correlate by flow cytometry (Fig. 5D; Supplementary Fig. S4D). Whereas levels of CCR7^+^ T cells could not discriminate between patients with long and short survival (Supplementary Fig. S4E and S4F), high CCR7^+^CD45RA^+^ naïve T cell or stem cell memory (50) levels trended toward being correlated to longer OS (Fig. 5E).

### Presence of Tertiary Lymphoid Structures as Correlates of Response and Survival

CCL21 expression in pretreatment tumors was associated with regions of the tumor which also contained T and B cells (refs. 51, 52; Supplementary Fig. S5A and S5B). These regions resembled tertiary lymphoid structures (TLS; refs. 53, 54) and will be referred to as TLS-like regions. CCR7^+^ cells also associated with TLS-like regions but because CD45RA was not costained it is conceivable that these cells were central memory T cells (Supplementary Fig. S5A–S5C).

**FIGURE 6:**
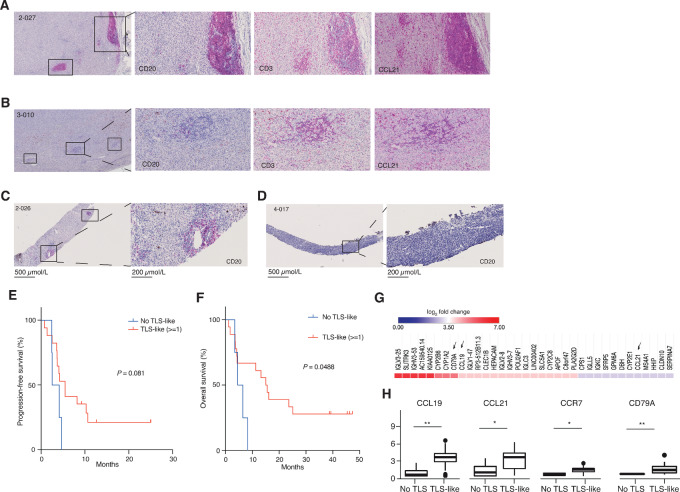
IHC magenta showing low mag of CD20 varied staining of TLS-like (borderline-tertiary lymphoid structures) followed by high magnification images of CD20, CD3, CCL21 within serial sections for Pt 2-027 (**A**), 3-010 (**B**). Also see Supplementary Fig. S5. Low and high magnification images of CD20^+^ TLS-like staining for Pt. 2-026 (**C**) and no TLS sample Pt. 4-017 (**D**). Kaplan–Meier analysis showing PFS (**E**) and OS (**F**), respectively dividing patient population into two groups based on IHC, no-TLS (*n* = 4) and TLS-like (*n* = 18). Also see Supplementary Fig. S5. **G,** Heatmap showing top 10% genes with highest log_2_ fold change, among all positively and negatively significantly regulated genes comparing no-TLS and TLS-like groups using bulk RNA-seq (log_2_ Reads Per Kilobase per Million mapped reads [RPKM] normalized values). Arrows represent relevant TLS-based gene signatures. Genes with FDR-adjusted *P* values <0.05 were considered statistically significant. **H,** Individual box plots showing *CCL19*, *CCL21*, *CCR7*, and *CD79A* changes in expression between the groups. Statistical tests were carried out using DESeq2 and FDR-adjusted *P* values were denoted with *, *P* < 0.05; **, *P* < 0.01; ***, *P* < 0.001.

To investigate how common TLS-like regions were in our biopsies, we performed staining with CD20 and CD3 antibodies, and scored tumors as either positive or negative for TLS-like regions. Most samples were positive for TLS-like regions (Fig. 6A–D). To assess whether the presence of TLS-like regions had any impact on survival, we dichotomized PFS and OS based on presence of TLS-like regions or not. Presence of TLS-like regions only trended to correlate with longer PFS but OS was significantly longer in patients whose tumors had TLS-like regions (Fig. 6E and F).

To assess whether there was any transcriptional changes between samples which had TLS-like regions and not, we analyzed the RNA-seq data from pretreatment biopsies (Fig. 6G). This analysis demonstrated higher levels of RNA encoding proteins involved in TLS, including B-cell and plasma cell genes encoding immunoglobulins, as well as *CD79A*, the chemokines *CCL19* and *CCL21*, and *CCR7* (Fig. 6H). These data suggest that TLS-like regions may play a role in immunotherapy effects in uveal melanoma. They correlate with CCL21 expression in TLS-like regions and the plasma levels correlate with survival. Interestingly though, whereas CCL21 and TLS-like regions correlate with survival, they did not correlate with gender of the patient or *BAP1* status. However, they negatively correlated with LDH levels (Supplementary Fig. S6A–S6D)

## Discussion

Treatment of melanoma with ICIs has been a mainstay in the clinic for almost a decade. However, many patients either do not respond to these therapies or they develop resistance or experience serious AEs requiring treatment cessation. Patients with uveal melanoma are generally unresponsive to immunotherapy, with the exception of rare cases where the tumors exhibit a high tumor mutational burden. This can be seen in uveal melanoma tumors that carry mutations in the gene encoding the DNA repair protein MBD4 (55, 56) or are of the iris subtype of uveal melanoma, as described recently (57, 58).

Studies so far show that combination therapies with PD-1 inhibitors and a CTLA4 inhibitor or an HDAC inhibitor lead to significantly higher numbers of patients with objective responses to therapy, compared with monotherapy using PD-1 inhibitors. However, despite this promising trend, current response rates are still low (22–24). Collectively, these results hint at the need of more research to identify and better understand the factors driving the observed resistance to ICIs in uveal melanoma. Only then can we hope to devise new treatments and trials for this patient group.

Analyses of blood and tissue correlates is useful to learn more about the dynamic changes of tumors during treatment and they can reveal candidate biomarkers that can be validated in other and larger studies. Previous analyses of tumors and blood from the PEMDAC trial suggested that genetics (*BAP1* status or a UV mutational signature), tumor burden (levels of ctDNA and LDH), and immune cell distribution (T cells, monocytes, and neutrophils) were associated with responses and survival (24). Here, we evaluated whether activity of TK1 in plasma could be used in uveal melanoma to monitor tumor responses. TK1 is a cytosolic protein involved in nucleotide biogenesis that leaks out from cancer cells when they die. TK1 has previously been shown to be a promising biomarker in patients treated with ICI (37, 59). High TK1 activity correlates with worse performance status, more advanced tumor stage and higher levels of LDH, another enzyme that leaks out of cancer cells. In this study, we found that TK1 levels varied during the course of the disease, which was not unexpected given that this was also observed with ctDNA in the PEMDAC study (24). However, none of the responders ever experienced levels higher than 300 DUA. Low (<150 DUA) mean TK1 activity throughout the study correlated with better survival. Therefore, in uveal melanoma, larger and additional studies, including multiple blood sampling, are needed to further evaluate TK1 as a potential biomarker.

Another outcome of the PEMDAC trials was the observation that treatment with pembrolizumab and entinostat resulted in increased levels of activated T cells and monocytes (24). In contrast, reductions in neutrophils were only observed in longer surviving patients (24). We found that levels of some of the factors were significantly different in pretreatment samples of different response groups, compared with samples from the 9-week treatment group or from the end-of-study group. We were intrigued to find that patients with PR exhibited higher levels of CCL21, compared with patients with PD. CCL21 is a chemokine that binds CCR7 on naïve T cells (42)**,** enabling them to enter into lymph nodes and get activated (49). In cancer, CCL21 can have additional functions, including altering the host immune response from immunogenic to tolerogenic by promoting the formation of lymphoid-like stromal components, which then impacts on tumor progression (39). Besides CCL21, we show that levels of chemokine CXCL13 was numerically higher in plasma from patients with PR in the PEMDAC trial, compared with patients with PD. Both CCL21 and CXCL13 can stimulate the formation of TLSs in tumors by recruitment of T cells, dendritic cells, and B cells (60–62). Indeed, in our study, we observed an increased expression of CCL21 in TLS-like structures. Notably though, this factor was regulated in the opposite direction during treatment in patients with SD or PD versus those with PR. It is tempting to speculate that the dual role of CCL21 in cancer could be represented by this pattern, where high levels is promoting TLS but a decrease in CCL21 can be favorable for therapy because that result in less stroma-mediated immune evasion (39). Interestingly, high CCL21 serum levels and presence of TLS-like regions in tumors negatively correlated with LDH levels. It is tempting to speculate that tumor burden may negatively impact the ability of TLS-like structures to form and that this is one of many reasons why larger tumors are harder to treat. The presented data on CCL21 and TLS-like regions in uveal melanoma is thus hypothesis generating and warrants further examination in future clinical and experimental studies.

Collectively, we present follow-up data from the PEMDAC trial which reveals durable responses to combination therapy in a small subset of patients. We make a first assessment of TK1 as a potential biomarker of tumor burden in uveal melanoma and report that combined pembrolizumab and entinostat therapy results in changes of cytokines and chemokines levels that are reflective of immune changes observed in the blood of the patients in the PEMDAC trial.

## Supplementary Material

Figure S1 Figure S1. a) Time To Response (TTR) according to RECIST 1.1. b) Duration of Response (DOR) according to RECIST 1.1. c) Quality of life assessments: Boxplot of EQ-5D VAS evaluation, screening and last known assessment at database lock. d) EQ-5D VAS, change from screening to last known assessment. e) The functional Assessment of Cancer Therapy – General (FACT-G) score sub-scales at screening and at last known assessments

Figure S2 Figure S2. a) Gene signatures implicating short term survival. Statistical tests were carried out using DESeq2 and FDR-adjusted P-values were denoted with *, P < 0.05; **, P < 0.01; ***, P < 0.001. b) Average of TK1 values assessed for all timepoints by the number of timepoints divided among response groups, PD, SD, PR. c) TK1 values in different response groups

Figure S3 Figure S3. a) Heat map showing plasma chemokine and cytokine levels pre-treatment to end of study (EOS) in patients with progressive disease (PD) b) Heat map of plasma chemokine levels pre-treatment to EOS in stable disease (SD) patients. c) Heat map of plasma chemokine levels pre-treatment to EOS in partial response (PR) patients d) Levels of CXCL9 in different response groups pretreatment and EOS. e) Levels of CXCL9 pretreatment and EOS in patients surviving longer or shorter in the PEMDAC trail.

Figure S4 Figure S4. Kaplan–Meier analysis showing PFS of a) CCL13 and b) IL-21. c) Flow cytometry plots showing CCR7 and CD45RA gating among CD3 positive cells in blood samples d) Flow cytometry based comparison between short (n=18) and long (n=6) term survivors with CD3+CCR7+ % (T naive, T stem cell memory and T central memory) analysis using pretreatment blood samples and e) Kaplan–Meier analysis showing Overall survival with median CD3+CCR7+ % (n=24).

Figure S5 Figure S5. Immunohistochemistry magenta of a) Immunohistochemistry magenta showing CCL21 and corresponding CCR7 expression within tumor biopsies for patient 1-013 and 3-009. b) Patient 1-013, a lymph node biopsy showing CD20, CD3 and CCL21 staining with serial sections. c) Patient 2-027, a liver met biopsy showing SOX10, CCR7, TK1 and PDL1 (DAB) within the same areas as shown in Fig 6.

Figure S6 Figure S6. Analysis of clinical parameters gender (a), tumor BAP1 status (b) and LDH levels (c-d) compared to serum levels of CCL21 (a-c) or presence of TLS-like regions in tumors.

Supplementary Table S1 Representativeness Table

Supplementary Table S2 Baseline characteristics, patient population

Supplementary Table S3 Patient sample information

Supplementary Table S4 Follow up adverse events (AE) of grade ≥3 (n = 29 patients)

Supplementary Table S5 Complete differential expression results

Supplementary Table S6 GSEA
