# Supplementary material for: Chemokine Analysis in Patients with Metastatic Uveal Melanoma Suggests a Role for CCL21 Signaling in Combined Epigenetic Therapy and Checkpoint Immunotherapy
Source: Cancer Res Commun. 2023 May 18;3(5):884–95. doi: 10.1158/2767-9764.CRC-22-0490 (PMC10194136; doi:10.1158/2767-9764.CRC-22-0490)
Supplement: Figure S1 — a) Time To Response (TTR) according to RECIST 1.1. b) Duration of Response (DOR) according to RECIST 1.1. c) Quality of life assessments: Boxplot of EQ-5D VAS evaluation, screening and last known assessment at database lock. d) EQ-5D VAS, change from screening to last known assessment. e) The functional Assessment of Cancer Therapy – General (FACT-G) score sub-scales at screening and at last known assessments [file crc-22-0490-s01.pdf]

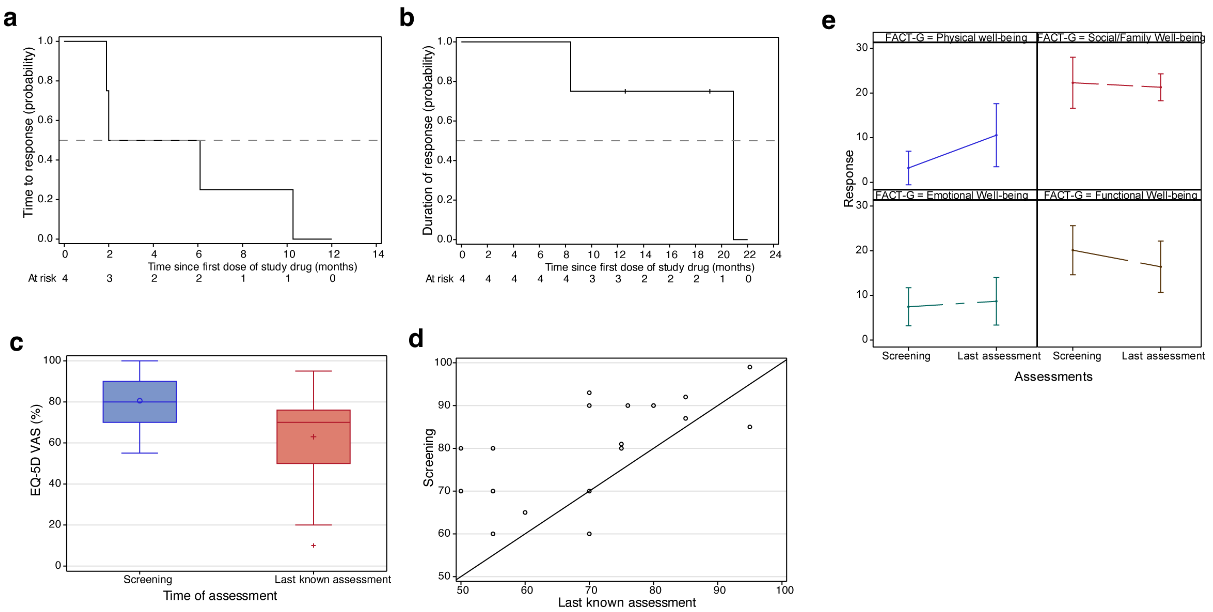

Figure S1. a) Time To Response (TTR) according to RECIST 1.1. b) Duration of Response (DOR) according to RECIST 1.1. c) Quality of life assessments: Boxplot of EQ-5D VAS evaluation, screening and last known assessment at database lock. d) EQ-5D VAS, change from screening to last known assessment. e) The functional Assessment of Cancer Therapy – General (FACT-G) score sub-scales at screening and at last known assessments.
