# Supplementary material for: Chemokine Analysis in Patients with Metastatic Uveal Melanoma Suggests a Role for CCL21 Signaling in Combined Epigenetic Therapy and Checkpoint Immunotherapy
Source: Cancer Res Commun. 2023 May 18;3(5):884–95. doi: 10.1158/2767-9764.CRC-22-0490 (PMC10194136; doi:10.1158/2767-9764.CRC-22-0490)
Supplement: Figure S2 — a) Gene signatures implicating short term survival. Statistical tests were carried out using DESeq2 and FDR-adjusted P-values were denoted with *, P < 0.05; **, P < 0.01; ***, P < 0.001. b) Average of TK1 values assessed for all timepoints by the number of timepoints divided among response groups, PD, SD, PR. c) TK1 values in different response groups [file crc-22-0490-s02.pdf]

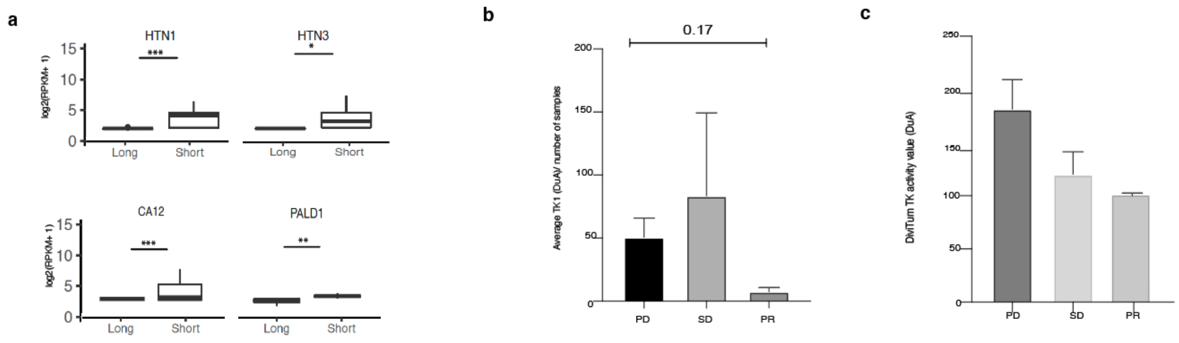

Figure S2. a) Gene signatures implicating short term survival. Statistical tests were carried out using DESeq2 and FDR-adjusted P-values were denoted with *, $P < 0.05$; **, $P < 0.01$; ***, $P < 0.001$. b) Average of TK1 values assessed for all timepoints by the number of timepoints divided among response groups, PD, SD, PR. c) TK1 values in different response groups.
