# Supplementary material for: Chemokine Analysis in Patients with Metastatic Uveal Melanoma Suggests a Role for CCL21 Signaling in Combined Epigenetic Therapy and Checkpoint Immunotherapy
Source: Cancer Res Commun. 2023 May 18;3(5):884–95. doi: 10.1158/2767-9764.CRC-22-0490 (PMC10194136; doi:10.1158/2767-9764.CRC-22-0490)
Supplement: Figure S3 — a) Heat map showing plasma chemokine and cytokine levels pre-treatment to end of study (EOS) in patients with progressive disease (PD) b) Heat map of plasma chemokine levels pre-treatment to EOS in stable disease (SD) patients. c) Heat map of plasma chemokine levels pre-treatment to EOS in partial response (PR) patients d) Levels of CXCL9 in different response groups pretreatment and EOS. e) Levels of CXCL9 pretreatment and EOS in patients surviving longer or shorter in the PEMDAC trail. [file crc-22-0490-s03.pdf]

Suppl Figure 3

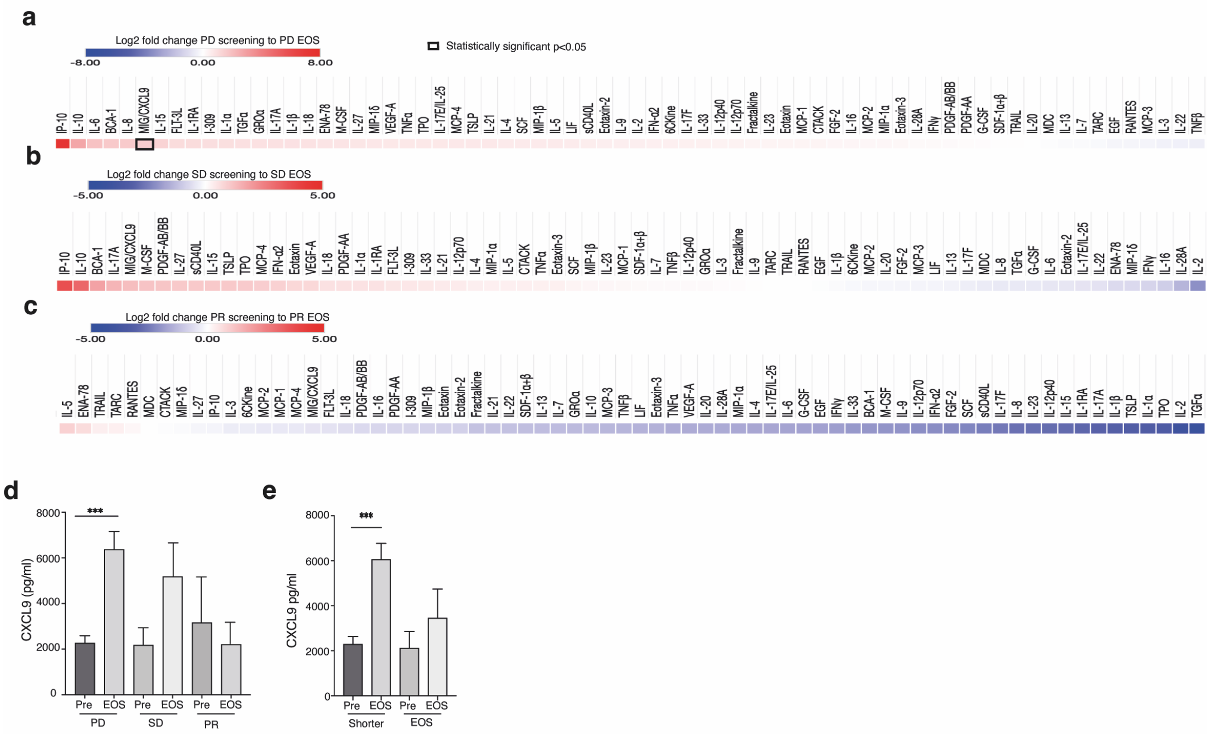

Figure S3. a) Heat map showing plasma chemokine and cytokine levels pre-treatment to end of study (EOS) in patients with progressive disease (PD) b) Heat map of plasma chemokine levels pre-treatment to EOS in stable disease (SD) patients. c) Heat map of plasma chemokine levels pre-treatment to EOS in partial response (PR) patients d) Levels of CXCL9 in different response groups pretreatment and EOS. e) Levels of CXCL9 pretreatment and EOS in patients surviving longer or shorter in the PEMDAC trail.
