# Supplementary material for: Chemokine Analysis in Patients with Metastatic Uveal Melanoma Suggests a Role for CCL21 Signaling in Combined Epigenetic Therapy and Checkpoint Immunotherapy
Source: Cancer Res Commun. 2023 May 18;3(5):884–95. doi: 10.1158/2767-9764.CRC-22-0490 (PMC10194136; doi:10.1158/2767-9764.CRC-22-0490)
Supplement: Figure S4 — Kaplan–Meier analysis showing PFS of a) CCL13 and b) IL-21. c) Flow cytometry plots showing CCR7 and CD45RA gating among CD3 positive cells in blood samples d) Flow cytometry based comparison between short (n=18) and long (n=6) term survivors with CD3+CCR7+ % (T naive, T stem cell memory and T central memory) analysis using pretreatment blood samples and e) Kaplan–Meier analysis showing Overall survival with median CD3+CCR7+ % (n=24). [file crc-22-0490-s04.pdf]

Suppl figure 4

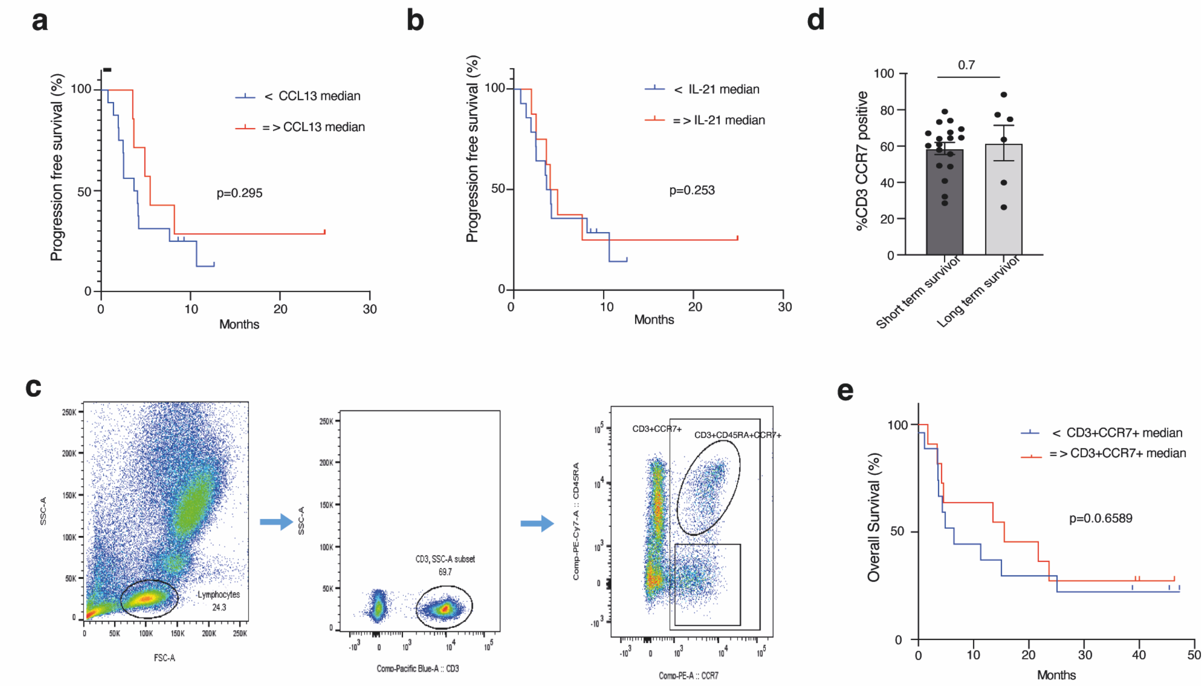

Figure S4. Kaplan–Meier analysis showing PFS of a) CCL13 and b) IL-21. c) Flow cytometry plots showing CCR7 and CD45RA gating among CD3 positive cells in blood samples d) Flow cytometry based comparison between short (n=18) and long (n=6) term survivors with CD3+CCR7+ % (T naive, T stem cell memory and T central memory) analysis using pre-treatment blood samples and e) Kaplan–Meier analysis showing Overall survival with median CD3+CCR7+ % (n=24).
