# Supplementary material for: Chemokine Analysis in Patients with Metastatic Uveal Melanoma Suggests a Role for CCL21 Signaling in Combined Epigenetic Therapy and Checkpoint Immunotherapy
Source: Cancer Res Commun. 2023 May 18;3(5):884–95. doi: 10.1158/2767-9764.CRC-22-0490 (PMC10194136; doi:10.1158/2767-9764.CRC-22-0490)
Supplement: Figure S5 — Immunohistochemistry magenta of a) Immunohistochemistry magenta showing CCL21 and corresponding CCR7 expression within tumor biopsies for patient 1-013 and 3-009. b) Patient 1-013, a lymph node biopsy showing CD20, CD3 and CCL21 staining with serial sections. c) Patient 2-027, a liver met biopsy showing SOX10, CCR7, TK1 and PDL1 (DAB) within the same areas as shown in Fig 6. [file crc-22-0490-s05.pdf]

Supple Figure 5

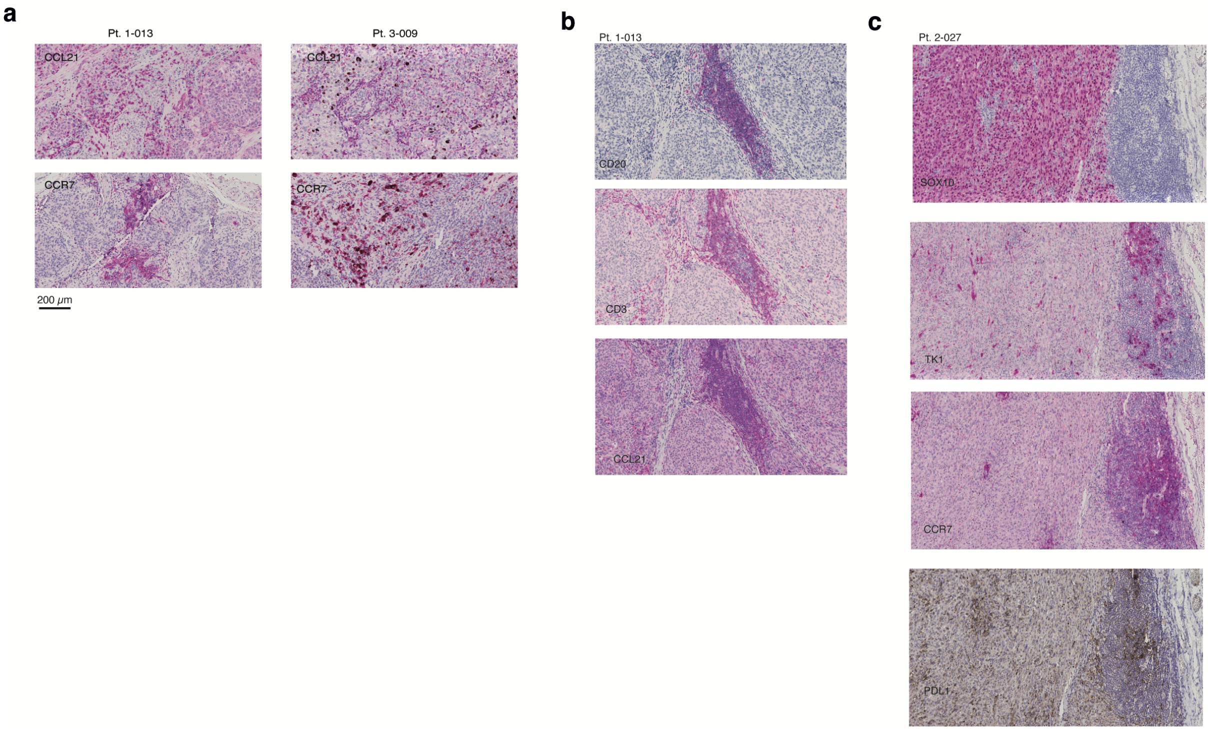

Figure S5. Immunohistochemistry magenta of a) Immunohistochemistry magenta showing CCL21 and corresponding CCR7 expression within tumor biopsies for patient 1-013 and 3-009. b) Patient 1-013, a lymph node biopsy showing CD20, CD3 and CCL21 staining with serial sections. c) Patient 2-027, a liver met biopsy showing SOX10, CCR7, TK1 and PDL1 (DAB) within the same areas as shown in Fig 6.
