# Supplementary material for: Chemokine Analysis in Patients with Metastatic Uveal Melanoma Suggests a Role for CCL21 Signaling in Combined Epigenetic Therapy and Checkpoint Immunotherapy
Source: Cancer Res Commun. 2023 May 18;3(5):884–95. doi: 10.1158/2767-9764.CRC-22-0490 (PMC10194136; doi:10.1158/2767-9764.CRC-22-0490)
Supplement: Figure S6 — Analysis of clinical parameters gender (a), tumor BAP1 status (b) and LDH levels (c-d) compared to serum levels of CCL21 (a-c) or presence of TLS-like regions in tumors. [file crc-22-0490-s06.pdf]

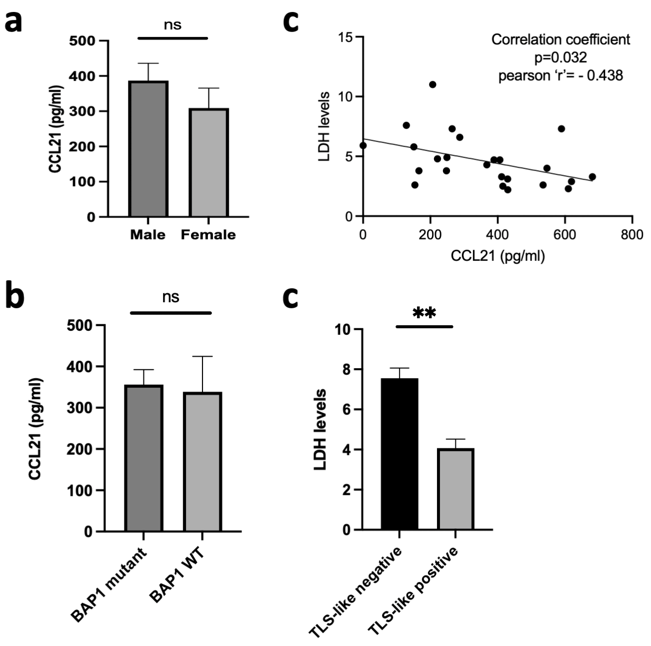

Figure S6. Analysis of clinical parameters gender (a), tumor BAP1 status (b) and LDH levels (c-d) compared to serum levels of CCL21 (a-c) or presence of TLS-like regions in tumors.
