# Supplementary material for: Chemokine Analysis in Patients with Metastatic Uveal Melanoma Suggests a Role for CCL21 Signaling in Combined Epigenetic Therapy and Checkpoint Immunotherapy
Source: Cancer Res Commun. 2023 May 18;3(5):884–95. doi: 10.1158/2767-9764.CRC-22-0490 (PMC10194136; doi:10.1158/2767-9764.CRC-22-0490)
Supplement: Supplementary Table S1 — Representativeness Table [file crc-22-0490-s07.pdf]

Supplementary Table 1. Representativeness of Study Participants

Cancer type(s)/subtype(s)/stage(s)/condition	Uveal melanoma (UM), stage IV (metastatic disease, AJCC 8th edition)
Considerations related to:	
Sex	Males have a 30% greater incidence than females.
Age	The median age at diagnosis of primary tumor of UM is 62 years. Development of metastatic disease occur in approximately 50% patients within the coming five years.
Race/ethnicity	In the US, the incidence is approximately five per million individuals, with a significantly higher incidence in non-Hispanic whites (6.02 per million) when compared with blacks and Asians (0.31 and 0.39 per million, respectively). Incidence in Hispanics is in the middle, at 1.67 per million.
Geography	In the US, the incidence is approximately five per million individuals.<br>In Europe, incidence increases with latitude, ranging from two per million in Spain and Italy, four to six per million in Central Europe, and greater than eight per million in Denmark and Norway. The incidence in South Korea is similar to Asians in the US, at 0.42 per million.<br>The incidence in Sweden is similar to that in Denmark and Norway, i.e. 8 per million with approximately 50% developing metastatic disease.
Other considerations	The study was carried out at four university hospitals with referrals from all Sweden. Enrollment of the 29 patients occurred February - December 2018.
Overall representativeness of this study	The patients included in the present study are all from Sweden with patient characteristics similar to what could be expected from epidemiological data in the literature, i.e. a median age of 70 years and 59% male and 41% female. The majority (90%) of patients had engagement of the liver, as expected.<br>12/29 patients had no previous treatment.

References

Krantz BA, Dave N, Komatsubara KM, Marr BP, Carvajal RD. Uveal melanoma: epidemiology, etiology, and treatment of primary disease. Clin Ophthalmol. 2017 Jan 31;11:279-289.

Rantala ES, Hernberg M, Kivelä TT. Overall survival after treatment for metastatic uveal melanoma: a systematic review and meta-analysis. Melanoma Res. 2019 Dec;29(6):561-568
