# Supplementary material for: Chemokine Analysis in Patients with Metastatic Uveal Melanoma Suggests a Role for CCL21 Signaling in Combined Epigenetic Therapy and Checkpoint Immunotherapy
Source: Cancer Res Commun. 2023 May 18;3(5):884–95. doi: 10.1158/2767-9764.CRC-22-0490 (PMC10194136; doi:10.1158/2767-9764.CRC-22-0490)
Supplement: Supplementary Table S2 — Baseline characteristics, patient population [file crc-22-0490-s08.pdf]

Supplementary Table 2. Baseline characteristics, patient population

**Baseline characteristics**	**Category**	**Pembrolizumab + Entinostat**
N		29
Age	Mean (SD)	68.1 (9.8)
	Median	70
	Min - Max	34 - 83
Gender, n(%)	Female	12 (41)
	Male	17 (59)
ECOG PS, n(%)	0	24 (83)
	1	5 (17)
Previous treatment for metastatic disease, n(%)*	Surgery	5 (17.2)
	Radiation therapy	1 (3)
	Chemotherapy	8 (28)
	Isolated Hepatic Perfusion	8 (28)
	No previous treatment	12 (41)
Time from metastatic disease until first dose of study drug (months)	Median	6.8
	Min to Max	-53.6 to -0.5
Disease range at start of treatment, n(%)	Liver only	10 (34)
	Extrahepatic only	3 (10)
	Liver and extrahepatic	16 (55)
LD, n(%)	>ULN	14 (48)
