# Supplementary material for: Chemokine Analysis in Patients with Metastatic Uveal Melanoma Suggests a Role for CCL21 Signaling in Combined Epigenetic Therapy and Checkpoint Immunotherapy
Source: Cancer Res Commun. 2023 May 18;3(5):884–95. doi: 10.1158/2767-9764.CRC-22-0490 (PMC10194136; doi:10.1158/2767-9764.CRC-22-0490)
Supplement: Supplementary Table S3 — Patient sample information [file crc-22-0490-s09.pdf]

Supplementary Table 3. Patient sample information

Supplementary Table 3. Patient sample information

Patient ID	Tumor Biopsy Site	2 yr followup responses	Genetics
1-001	Liver	Short-term	BAP1 Mutant
1-002	Liver	Short-term	BAP1 Mutant
1-003	Liver	Short-term	BAP1 Mutant
1-004	Liver	Long-term	BAP1 WT
1-005	Liver	Short-term	BAP1 Mutant
1-006	Liver	Short-term	BAP1 Mutant
3-007	Liver	Short-term	BAP1 Mutant
2-008	Liver	Long-term	BAP1 Mutant
3-009	Liver	Short-term	BAP1 Mutant
3-010	Liver	Long-term	BAP1 Mutant
1-011	Liver	Short-term	BAP1 Mutant
3-012	Liver	Short-term	BAP1 WT
1-013	Lymph node	Short-term	BAP1 WT
3-014	Liver	Short-term	BAP1 Mutant
1-015	Liver	Short-term	BAP1 Mutant
3-016	Liver	Short-term	BAP1 Mutant
4-017	Liver	Short-term	BAP1 Mutant
2-018	Liver	Long-term	BAP1 Mutant
4-019	Lymph node	Short-term	BAP1 Mutant
3-020	Liver	Short-term	BAP1 Mutant
1-021	Liver	Short-term	BAP1 Mutant
4-022*	Liver	Long-term	BAP1 Mutant
2-023	Liver	Short-term	BAP1 Mutant
2-024	Liver	Short-term	BAP1 Mutant
1-025	Subcutaneous	Short-term	BAP1 Mutant
2-026	Liver	Short-term	BAP1 WT
2-027	Liver	Long-term	BAP1 WT
4-028	Liver	Short-term	BAP1 Mutant
1-029	Liver	Long-term	BAP1 WT
* Iris Melanoma			
