# Supplementary material for: Chemokine Analysis in Patients with Metastatic Uveal Melanoma Suggests a Role for CCL21 Signaling in Combined Epigenetic Therapy and Checkpoint Immunotherapy
Source: Cancer Res Commun. 2023 May 18;3(5):884–95. doi: 10.1158/2767-9764.CRC-22-0490 (PMC10194136; doi:10.1158/2767-9764.CRC-22-0490)
Supplement: Supplementary Table S4 — Follow up adverse events (AE) of grade ≥3 (n = 29 patients) [file crc-22-0490-s10.pdf]

Supplementary Table 4. Follow up adverse events (AE) of grade ≥3 (n = 29 patients)

		AE of severity grade ≥ 3 and treatment related to				
**System Organ Class**	**Preferred Term**	**Entinostat n (%)**	**Pembrolizumab n (%)**	**Entinostat+ Pembrolizumab n (%)**	**Other n (%)**	**Total n (%)**
Per Protocol population						29
Patients with any Treatment Related Adverse Event and severity ≥ 3		6 (20.7)	6 (20.7)	5 (17.2)	3 (10.3)	18 (62.1)
Blood and lymphatic system disorders		4 (13.8)				4 (13.8)
	Lymphopenia	1				1
	Neutropenia	3				3
Endocrine disorders			1 (3.4)			1 (3.4)
	Hypophysitis		1			1
Gastrointestinal disorders		1 (3.4)		2 (6.9)		3 (10.3)
	Colitis			1		1
	Nausea	1				1
	Stomatitis			1		1
Hepatobiliary disorders					1 (3.4)	1 (3.4)
	Jaundice				1	1
Investigations			5 (17.2)	1 (3.4)		7 (24.1)
	Alanine aminotransferase increased		2			2
	Aspartate aminotransferase increased		3			3
	Cortisol decreased		1			1
	Blood alkaline phosphatase increased		3	1		4
Metabolism and nutrition disorders		1 (3.4)		1 (3.4)	1 (3.4)	3 (10.3)
	Hyperglycaemia	1				1
	Hypokalaemia				1	1
	Hyponatraemia			1		1
Musculoskeletal and connective tissue disorders						1 (3.4)
	Lupus-like syndrome			1		1
Respiratory, thoracic and mediastinal disorders					1 (3.4)	1 (3.4)
	Pulmonary embolism				1	1
Skin and subcutaneous tissue disorders				1 (3.4)		2 (6.9)
	Rash			1		1
	Rash papular			1		1
